# Epithelial-mesenchymal transition and apoptosis of renal tubular epithelial cells are associated with disease progression in patients with IgA nephropathy

**DOI:** 10.3892/mmr.2014.2179

**Published:** 2014-04-24

**Authors:** JUNXIA YAO, ZUNJI KE, XIAOYAN WANG, FENG PENG, BIN LI, RENLIANG WU

**Affiliations:** 1 Institute of Pathology, Tongji Medical College, Huazhong University of Science and Technology, Wuhan, Hubei 430030, P.R. China; 2 Department of Pathology, Qingpu Branch of Zhongshan Hospital Affiliated to Fudan University, Shanghai 201700, P.R. China; 3 Department of Pathology, Hubei University of Medicine, Shiyan, Hubei 442000, P.R. China

**Keywords:** renal tubular epithelial cells, epithelial-mesenchymal transition, apoptosis, prognosis, Immunoglobulin A nephropathy

## Abstract

The aim of the present study was to examine the effects of epithelial-mesenchymal transition (EMT) and apoptosis of renal tubular epithelial cells on the prognosis of immunoglobulin A (IgA) nephropathy. Renal biopsy tissues from 74 cases of IgA nephropathy were divided into a mild mesangial proliferation group (27 cases), a focal hyperplasia group (28 cases) and a proliferative sclerosis group (19 cases). The blood pressure, serum creatinine and 24 h urinary protein excretion of all patients were detected. To define EMT, α-smooth muscle actin (α-SMA), vimentin and collagen fibers were assessed. Apoptosis was determined by terminal deoxynucleotidyl transferase dUTP nick end labeling (TUNEL). The blood pressure, serum creatinine and 24 h urinary protein excretion of patients with IgA nephropathy altered with increasing pathological grade. All clinical indices of patients in the proliferative sclerosis group were higher than those of the other two groups, and the 24 h urinary protein excretion of the focal hyperplasia group was statistically higher than that of the mild mesangial proliferation group. The expression of tubular interstitial α-SMA, vimentin and collagen fibers increased with the pathological grade and was closely correlated with clinical indices, including collagen fibers and 24 h urinary protein excretion. TUNEL-positive cells increased with the exacerbation of pathological changes. The EMT and apoptosis of renal tubular epithelial cells reflected the clinical severity of IgA nephropathy. α-SMA, vimentin and the apoptotic index may be used as important markers for evaluating the prognosis of IgA nephropathy.

## Introduction

Immunoglobulin A (IgA) nephropathy is also termed chronic glomerulonephritis and is characterized by deposits of IgA in the mesangial areas of the renal glomeruli and by recurring hematuria with only partial proteinuria. It occurs more commonly in young male adults. The clinical and pathological alterations of IgA nephropathy are diverse. Patient prognosis was initially considered to be good; however, several studies revealed that 10 years after the incidence of IgA nephropathy, 15–40% of patients enter the end-stage of renal failure (1,2).

Tubulointerstitial fibrosis is an important factor affecting the development and prognosis of IgA nephropathy (3). Previous studies have demonstrated that renal tubular epithelial-mesenchymal transition (EMT) was an important mechanism in tubulointerstitial fibrosis (4,5). When EMT occurs, renal tubular epithelial cells lose the characteristics of epithelial cells and the expression of α-SMA and vimentin, which is usually expressed in mesenchymal cells. The transformed cells are functionally similar myofibroblasts (MF), which synthesize the extracellular matrix (ECM) and contribute to the progressive development of renal interstitial fibrosis (6,7). Apoptosis of renal tubular cells is also known to contribute to the regulation of the renal cell number with an induction and repair of renal injury. The apoptosis of proximal tubular cells also occurs during renal tubular epithelial cell-mesenchymal transformation (8). EMT and apoptosis are major mechanisms of renal injury; however, there have been few studies investigating the effects of EMT and apoptosis of renal tubular epithelial cells on the prognosis of IgA nephropathy. In the present study, the renal biopsy sample tissues with IgA nephropathy were analyzed using immunohistochemical terminal deoxynucleotidyl transferase dUTP nick end labeling (TUNEL) and Masson staining. The correlation between the transdifferentiation and apoptosis of renal tubular epithelial cells and clinical prognosis was analyzed.

## Materials and methods

### Samples and patients

In total, 74 renal biopsies with IgA nephropathy were selected, including 32 male cases and 42 female cases aged between 14 and 51 years (average age, 27.86±9.73 years), and cases with purpura nephritis, HBV-associated glomerulonephritis and lupus nephritis were excluded. The histological grading was based on Lee’s grading (9) and IgA nephropathy was further divided into the following groups: A, the mild mesangial proliferative group (Lee grade: level I–II, 27 cases); B, the focal hyperplasia group (Lee grade: level III, 28 cases); and C, the proliferative sclerosis group (Lee grade: level IV–V, 19 cases). Three normal renal tissue samples far from tumors on nephrectomy biopsies were selected as the control (9). All experiments were performed in the Laboratory of Medical Biomechanics, Hubei University of Medicine (Shiyan, China). The samples were pathologically confirmed by Taihe Hospital of Hubei, University of Medicine (Shiyan, China). The Institutional Ethics Committee of Hubei University of Medicine (Shiyan, China) approved the project and all the patients gave informed consent prior to participation.

### Staining of collagen fibers

Collagen fibers in samples of renal needle biopsy and normal control samples were detected by Masson trichrome staining (Masson trichrome kit; Fuzhou Maixin Biotechnology Co., Ltd., Fuzhou, Fujian, China) with collagen fibers staining blue. Under the microscope, the ratio between renal interstitial areas with blue staining and total areas in the same visual field was calculated.

### Immunohistochemical staining

The samples were fixed in 4% paraformaldehyde and embedded in paraffin. The sections were cut into 4-μm thick slices from paraffin blocks. Following deparaffinization and rehydration, the sections were boiled with citrate buffer for antigen retrieval. Following washing, they were immersed in 3% H_2_O_2_ in methanol to inhibit endogenous peroxidase. Anti-α-SMA and anti-vimentin (Fuzhou Maixin Biotechnology Co., Ltd.) were applied as primary antibodies. Phosphate-buffered saline (PBS) was used to replace the primary antibody in the negative controls and then the sections were incubated at 4°C overnight. The biotin-labeled secondary antibody and streptavidin-peroxidase were also added drop by drop, with diaminobenzidine (DAB) color development. All the reagents were purchased from Fuzhou Maixin Biotechnology Co., Ltd..

The Motic Med 6.0 pathological image analysis software from Beijing University of Aeronautics and Astronautics (Beijing, China) was selected to collect five visions (magnification, ×400) for each sample. The integrated optical density of tubulointerstitial α-SMA and vimentin-positive areas was examined and the average value was obtained.

### Detection of apoptotic cells by TUNEL

The TUNEL kit purchased from Boehringer Mannheim GMBH (Mannheim, Germany) was used to label the apoptotic cells. The paraffin sections were processed by conventional dewaxing to water and the proteinase K (mass concentration of proteinase K was 20 mg/l and 10 mmol/l in Tris-HCl; pH 8.0) was then added for 15–25 min of digestion at 37°C. The sections were then washed in PBS and the methanol solution of H_2_O_2_ with the mass concentration of 3 g/l was used for sealing, which was followed by storing for 30 min at room temperature. Following this, the sections were washed in PBS, TUNEL reaction solution was added, the negative and positive controls were established and they were incubated for 30 min at 37°C. Then, the steps including washing in PBS, adding the conversion agent-POD and incubation for 30 min at 37°C were conducted. Following which, the sections were washed in PBS and DAB staining was performed for 3 min. In addition, distilled water was selected for washing and hematoxylin was selected for re-staining. Finally, dehydration, transparency processing and observation using a light microscope (BX51; Olympus Optical Co., Ltd. Tokyo, Japan) were performed. The cells with brown particles in the nucleus were the apoptotic cells.

Under a high-power microscope (magnification, ×400), five visions were randomly collected and analyzed by pathological image software (Motic Med 6.0; Beijing University of Aeronautics and Astronautics), and the positive cells were counted, with the average value of five visions as the index of cell apoptosis.

### Statistical analysis

Data are shown as the mean ± standard error. The SPSS 13.0 software (SPSS, Inc., Chicago, IL, USA) was used for statistical analysis. Statistical analyses were performed using analysis of variance and linear trend. Individual comparisons were made using Fisher’s least significant difference. P<0.05 was considered to indicate a statistically significant difference.

## Results

### Clinical indicators

The blood pressure, serum creatinine and 24 h urinary protein excretion of patients with IgA nephropathy in three groups were analyzed. The clinical indices of the proliferative sclerosis group were significantly higher than those of the mild proliferation group and focal hyperplasia group (P<0.01). All clinical indices of the focal hyperplasia group were higher than those of the mild proliferation group, while the difference in 24 h urinary protein excretion between these two groups was statistically significant (P<0.01; Table I).

### Pathological indices

#### Apoptosis

**Figure 1:**
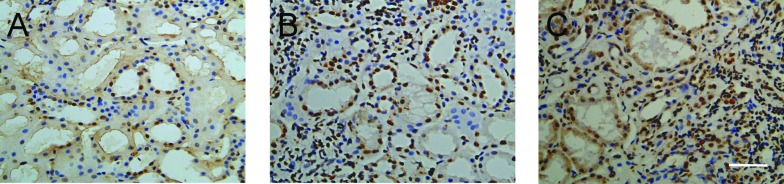
(A) Apoptotic cell death in IgA nephropathy. TUNEL-positive cells occasionally appeared in normal renal tissue and TUNEL-positive cells occurred in tubules and interstitium of tissues with a (B) single and (C) scattered distribution in IgA nephropathy. Scale bar, 50 μm. TUNEL, terminal deoxynucleotidyl transferase dUTP nick end labeling; IgA; immunoglobulin A.

TUNEL-positive cells were distributed in tubules and the interstitium of IgA nephropathy tissue, with single, scattered or small cluster-like models, when those cells were rare in normal renal tissue (Fig. 1). The tubulointerstitial apoptotic index of IgA nephropathy in different pathological grades increased significantly (P<0.01; Table II).

### Expression and distribution of α-SMA

**Figure 2:**
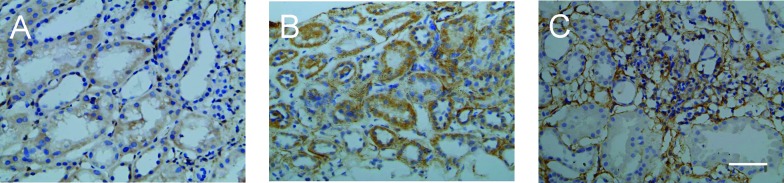
Expression of α-SMA in IgA nephropathy. (A) α-SMA was only expressed in vascular smooth muscle cells in normal renal tissue. (B) Expression of α-SMA increased in renal tubular epithelial cells and renal interstitial cells (C) and renal tubular disorders occurred in the proliferative sclerosis group in IgA nephropathy. Scale bar, 50 μm. α-SMA, α-smooth muscle actin; IgA, immunoglobulin A.

In normal renal tissue, α-SMA staining was positive only in vascular smooth muscle cells and not present in glomerular and tubulointerstitial cells. In IgA nephropathy, α-SMA expression was increased in renal tubular epithelial cells and renal interstitial cells (Fig. 2), and without changes in the glomerulus. Quantitatively, the expression of α-SMA increased in tubulointerstitial cells in hyperplasia and the proliferative sclerosis groups compared with that in the mild mesangial proliferation group (Table II).

### Expression and distribution of vimentin

**Figure 3:**
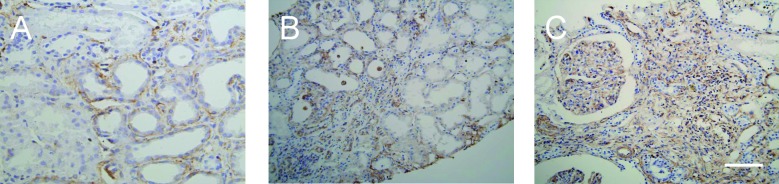
Expression of vimentin in IgA nephropathy. (A) Vimentin was expressed in glomerular mesangial cells and certain renal interstitial cells in normal renal tissue; however, the renal tubular epithelial cells and (B) vimentin-positive cells occurred in the glomerulus. (C) Expression increased in renal tubular epithelial cells and in renal interstitial cells in IgA nephropathy. Scale bar, 50 μm. IgA, immunoglobulin A.

Vimentin was expressed in glomerular mesangial and certain renal interstitial cells of normal renal tissue; however, it was not expressed in renal tubular epithelial cells. In IgA nephropathy tissue, vimentin levels were higher in the glomerulus and expressed in renal tubular epithelial and interstitial cells (Fig. 3). Quantitative data demonstrated that the tubulointerstitial expression of vimentin was different in groups with different pathological grades (P<0.05 or P<0.01; Table II).

### Expression of collagen fibers

**Figure 4:**
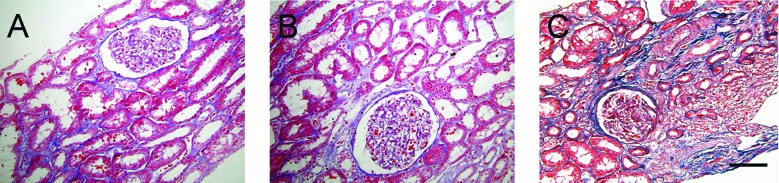
Expression of collagen fibers. (A) Masson staining shows collagen fibers (blue) in the renal interstitium. (B and C) Expression of interstitial collagen fibers increased in the renal interstitium of immunoglobulin A nephropathy with pathological grading. Scale bar, 50 μm.

Masson staining showed collagen fibers in the renal interstitium. Interstitial collagen fibers increased in the renal interstitium of patients with IgA nephropathy with increasing pathological grading (Fig. 4). Quantitative data demonstrated that interstitial collagen fibers increased more in the proliferative sclerosis group than those in the mild proliferation group and the focal hyperplasia group (P<0.01), and those in the focal hyperplasia group were higher than those in the mild proliferation group (P<0.01; Table II).

### Relevance between the expression of vimentin, α-SMA and collagen fibers and serum creatinine and 24 h urinary protein excretion

Statistical analysis demonstrated that serum creatinine and 24 h urinary protein excretion was moderately correlated with the apoptotic index, expression of vimentin, α-SMA and collagen fibers (0.4≤r≤0.7; P<0.01; Table III).

## Discussion

IgA nephropathy is a chronic renal disease and is the most common form of primary glomerular disease in the world, with ~20–40% of patients eventually entering into the end-stage of renal failure within 10–20 years (1–3,10). Currently, the most common clinical features of IgA nephropathy include blood pressure, serum creatinine and 24 h urinary protein excretion, which are closely correlated with histological grading; however, they are not associated with the renal prognosis (11,12). To further verify the association of blood pressure, serum creatinine and 24 h urinary protein excretion with the histological pathological alterations in patients with IgA nephropathy, previous studies grouped the patients according to the pathological diagnosis of renal biopsy tissue, which is different from the Oxford classification, by detailing the immunohistology of IgA nephropathy (13,14). The present study grouped the patients by a modification of Lee’s method, in which IgA nephropathy was further divided into the following groups: The mild mesangial proliferative group (Lee grade: level I–II), the focal hyperplasia group (Lee grade: level III) and the proliferative sclerosis group (Lee grade: level IV–V) (9). The results demonstrated that blood pressure, serum creatinine and 24 h urinary protein excretion of patients with IgA nephropathy worsened with increasing grade. Each clinical indicator of patients in the proliferative sclerosis group was significantly higher than that in the mild proliferation and local hyperplasia groups (P<0.01). In addition, 24 h urinary protein excretion of the focal hyperplasia group was significantly higher than that of the mild proliferation group (P<0.01). Collagen fibers, indicating the degree of fibrosis between different pathological grading, also ascended as the clinical stages gradually increased. Collagen fibers in the proliferative sclerosis group significantly deteriorated compared with those in the mild proliferation and local hyperplasia groups (P<0.01), and apparently increased in the focal hyperplasia group compared with the mild proliferation group (P<0.01). Serum creatinine and 24 h urinary protein excretion of patients were interrelated with the expression of collagen fibers (P<0.01). These results confirmed that the degree of interstitial fibrosis gradually worsened with the different pathological grading of IgA nephropathy, and proteinuria in patients increased, renal function weakened and the prognosis gradually deteriorated.

The main pathological symptoms of renal tubulointerstitial fibrosis include renal interstitial ECM accumulation, renal tubular atrophy and increased MFs in the renal interstitium. The pivotal step in renal interstitial fibrosis is the activation of fibroblasts, whose activated form is MFs. The quantity of collagen secreted by MFs is four- to five-fold higher compared with that secreted by fibroblasts, exacerbating ECM extracellular deposition and leading to renal structural remodeling due to its strong capacity to contract. Under pathological conditions, renal tubular epithelial cells possessed the capacity to transform into mesenchymal cells. In 1995, it was reported for the first time by Strutz *et al* (15) that EMT appears in renal fibrosis, followed by studies by Okada *et al* (16) and Fan *et al* (17), respectively, reporting that mice and rat renal tubular epithelial cells are able to transdifferentiate into MFs in experiments *in vitro*. Additionally, analysis of renal biopsies of 133 patients with different types of nephropathy revealed that the quantity of renal tubular epithelial cells with EMT characteristics was closely associated with the concentration of serum creatinine, as well as the degree of renal interstitial damage (18), suggesting that EMT is involved in the process of renal fibrosis (15). In the present study, it was observed that α-SMA and vimentin were expressed in renal tubular epithelial cells of IgA nephropathy renal biopsies. Among IgA nephropathy with different pathological grades, with the development of the disease, tubulointerstitial α-SMA and vimentin expression gradually increased (P<0.05 or P<0.01). This suggested that the phenotypic transformation indeed occurred in renal tubular epithelial cells of IgA nephropathy and, with the development of IgA nephropathy, transdifferentiation of tubule epithelial cells gradually increased. In addition, renal tubulointerstitial α-SMA and vimentin expression correlated with the expression of collagen fibers (P<0.01), and closely correlated with serum creatinine and 24 h urinary protein excretion of patients (P<0.01). These results lead to the conclusion that transdifferentiation of tubule epithelial cells is involved in the progression of the renal interstitial fibrosis lesion, relevant to the alterations in serum creatinine and 24 h urinary protein excretion of patients. Accordingly, it was verified that in IgA nephropathy, the transdifferentiation degree of renal tubular epithelial cells is associated with renal and tubulointerstitial fiber dysfunction, and the transdifferentiation of renal tubular epithelial cells may be one of the key factors leading to poor renal prognosis. Certainly, the detection of α-SMA and vimentin may be used as one of the clinical indicators in the assessment of IgA nephropathy prognosis.

Renal interstitial fibrosis lesions are usually paralleled with renal tubular atrophy, due to the involvement of renal tubular epithelial cells in the process of renal interstitial fibrosis through apoptosis. The present study showed that, in IgA nephropathy, apoptosis can be detected mainly in renal tubules and interstitium, particularly at sites where MFs have infiltrated, which is consistent with previous studies (16). It was also revealed that apoptosis in tissues increased with the development of the lesion. The apoptotic index was relatively low in the mild proliferation group and the highest in the focal hyperplasia group and decreased slightly in the proliferative sclerosis group (P<0.01). Therefore, it is hypothesized that at the early stages of lesions, the rate of apoptosis was relatively low, and as the lesion developed, apoptosis of tubule epithelial cells and interstitial cells increased. However, at the end-stage of lesions, the majority of tubule atrophy and the involvement of fibrosis in a relatively large area reduced the amount of apoptosis. Furthermore, it was also demonstrated that the apoptotic index of the tubulointerstitial region was moderately correlated with the degree of expression of interstitial MFs, fibrosis and clinical prognosis (P<0.01). In conclusion, tubulointerstitial cell apoptosis may be one of the factors leading to a poor prognosis in IgA nephropathy.

The present study demonstrated that α-SMA and vimentin expression of tubule epithelial cells, interstitial MF accumulation, collagen deposition, apoptosis of tubular epithelial and interstitial cells, interstitial fibrosis and renal dysfunction have a significant relevance in IgA nephropathy. Thus, it may be concluded that renal tubular epithelial cells in IgA nephropathy generate MFs through transdifferentiation, secreting a large quantity of collagen and finally leading to fibrosis, apoptosis of certain tubular epithelial cells, tubular atrophy and renal failure. Therefore, the transdifferentiation and apoptosis of renal tubular epithelial cells reflected the clinical severity of IgA nephropathy, and the detection of the expression of α-SMA, vimentin and apoptosis may be useful as an important index for evaluating the prognosis of IgA nephropathy.

## Figures and Tables

**Table I:** Clinical pathological features of immunoglobulin A nephropathy.

Group	Cases (n)	SBP (mm Hg^c^)	DBP (mm Hg)	Scr (μmol/l)	Upro (g/24 h)
Mild mesangial proliferation (A)	27	117.85±14.81	74.30±7.29	70.70±13.21	0.49±0.60
Hyperplasia (B)	28	124.82±16.74	77.93±10.28	95.22±25.41	1.70±1.60^a^,^b^
Proliferative sclerosis (C)	19	141.74±31.67^a^,^b^	94.47±19.37^a^,^b^	150.11±109.80^a^,^b^	2.86±1.65^a^,^b^

Data are presented as the mean ± standard deviation.

a P*<*0.01, compared with A;

b P*<*0.01, compared with B;

c 1 mm Hg=0.133 kpa.

SBP, systolic blood pressure; DBP, diastolic blood pressure; Scr, serum creatinine; Upro, urinary protein.

**Table II:** Tubulointerstitial cell apoptotic index and expression of α-SMA, vimentin and collagen fibers in immunoglobulin A nephropathy in different pathological grades.

Group	Cases (n)	Apoptotic index	α-SMA	Vimentin	Collagen fibers
Mild mesangial proliferation (A)	27	18.08±5.31	8.43±3.09	9.79±3.25	12.51±3.46
Hyperplasia (B)	28	73.52±12.25^a^	15.29±3.55^a^	17.05±3.51^a^	24.47±6.44^a^
Proliferative sclerosis (C)	19	48.79±11.06^a^,^c^	13.13±3.66^a^,^b^	19.50±4.13^a^,^b^	31.16±6.43^a^,^c^

Data are presented as the mean ± standard deviation.

a P<0.01, compared with A;

b P<0.05, compared with B;

c P<0.01.

α-SMA, α-smooth muscle actin.

**Table III:** Relevance between the expression of apoptotic indexes, vimentin, α-SMA and collagen fibers in immunoglobulin A nephropathy tissue (relevance coefficient r; n=74).

Indeces	Apoptotic index	α-SMA	Vimentin	Collagen fibers
Creatinine	0.521^a^	0.408^a^	0.533^a^	0.529^a^
Urinary protein	0.457^a^	0.466^a^	0.548^a^	0.574^a^

a P<0.01, pairwise comparison.

α-SMA, α-smooth muscle actin.
